# Hot topics in Cellular Neuropathology IV—targeting neurological diseases by gene therapy approaches

**DOI:** 10.3389/fncel.2025.1769815

**Published:** 2026-01-06

**Authors:** Dirk M. Hermann

**Affiliations:** Department of Neurology, University Hospital Essen, University of Duisburg-Essen, Essen, Germany

**Keywords:** adeno-associated viral, CRISPR/Cas9 technology, gene editing, gene therapy, neurodegenerative disease, neurodevelopment, small interfering RNA

In 1953, James Watson (1928–2025) has made the seminal discovery of the helical structure of DNA together with Francis Crick (1916–2004), a breakthrough that revealed how genetic information is stored and replicated in living organisms (Watson and Crick, 1953). For their discovery that DNA consists of two complementary, antiparallel strands held together by specific base-pairing, both researchers were awarded the Nobel prize in Physiology or Medicine in 1962. This insight laid the foundation for modern molecular biology, enabling further understanding of gene expression, heredity, and mutation.

The work of Charpentier and Jennifer A. Doudna in 2012 transformed genetic engineering by introducing a method for incorporating gene sequences into genomes with exceptional precision and efficiency (Jinek et al., 2012). The discovery of CRISPR/Cas9 emerged from research on *Streptococcus pyogenes*, in which Charpentier identified tracrRNA, a previously unknown molecule that plays a critical role in the bacterium's innate antiviral defense by enabling the cleavage of viral DNA (Deltcheva et al., 2011). Through targeted modification of the bacterium's molecular and enzymatic systems, Charpentier and Doudna engineered these molecular scissors to cleave DNA at predetermined sites (Jinek et al., 2012). This technique has revealed unprecedented possibilities in gene editing. For their discovery, both scientists received the Nobel Prize in Chemistry in 2020.

Gene editing and therapy have evolved as a pillar stone of modern biotechnology in the last 10 years. Its utilization across biological sciences emphasizes its importance and relevance, and it applications have extended far into the field of clinical medical sciences, including clinical neurology. More specifically, gene editing and therapy has inseminated Cellular Neuropathology as follows:

• Neurodegenerative disease treatment: Gene therapy technology holds promise for inherited neurodegenerative disease treatment. Its potential for correcting mutations associated with neurodegenerative diseases, such as spinal muscular atrophy (SMA) (Mendell et al., 2017; Day et al., 2021) and Huntington's disease (Morelli et al., 2023; Dolgin, 2025), is currently being explored. In randomized controlled studies, gene therapy approaches revealed striking therapeutic efficacy in human patients in disease areas, which so far seemed untreatable (Mendell et al., 2017; Day et al., 2021). Thereby, gene therapy entered clinical practice.
• Neurological disease modeling: The application of gene-editing approaches, including CRISPR/Cas9, has facilitated the development of cellular and animal models of neurological diseases, thereby enhancing the study of disease mechanisms and the identification of underlying pathological processes in disorders such as Alzheimer's disease (Schrauben et al., 2020), Parkinson's disease (Yang et al., 2019) and autism (Elamin et al., 2023).
• Understanding neural development: Through precise gene manipulation during development, scientists have gained insight into the roles of individual genes in shaping the brain (Zhang et al., 2018). This knowledge can provide insight into a range of developmental disorders. Three-dimensional induced pluripotent stem cell (iPSC) organoids have recently attracted great interest in the analysis of human brain development and pathology (Jain et al., 2025). Human brain organoids offer unprecedented possibilities for evaluating gene therapies during neural development and under neurodegenerative conditions.
• Functional genomics: The application of gene technologies has made it possible to conduct large-scale functional genomics investigations in neural cells (Sandberg et al., 2018), thereby enabling the identification of genes central to neural function and connectivity and improving understanding of conditions linked to abnormal neural network activity, including epilepsies.
• Regenerative medicine: Gene-editing technologies have been explored for their potential to promote neural regeneration following central nervous system injuries (Keatinge et al., 2021). Targeted gene editing is anticipated to enhance the repair of damaged neural tissue.

In an effort to highlight the most promising advances in translational neuroscience, the Cellular Neuropathology section of Frontiers in Cellular Neurosciences recently launched the Hot Topics hub (Hermann, 2020). This platform seeks out impactful papers that have the potential to reshape the field, expand conceptual horizons, and enhance current diagnostic or therapeutic approaches. Previous Research Topics within the hub have examined the role of subtle neuroinflammation in chronic neurodegeneration (Hermann et al., 2022), the contribution of neuronal plasticity to CNS repair (Hermann et al., 2023), and the application of gene editing in understanding neurological diseases (Hermann, 2023).

In commemoration of James Watson's recent death on November 6, 2025, the Hot Topics platform of the Cellular Neuropathology section of Frontiers in Cellular Neuroscience would like to shift the focus from distinct disease mechanisms and biological processes to a therapeutic strategy, which broadly transforms several neurodegenerative diseases at this moment, that is, gene therapy. As in the previous Research Topics, suitable manuscripts should push our understanding of neurological diseases, overcome existing limitations, pave the way for therapeutic progress and identify questions that deserve attention in future studies. This Research Topic is open for research in all disease areas. Papers outlining limitations and challenges, including ethical implications, of gene therapy are particularly invited. This Research Topic aims to attract Original research, Review, Perspective and Opinion manuscripts. Papers will be reviewed based on excellence, originality and innovation potential. Outstanding papers will be featured in an editorial. We are looking forward to your contribution to this new Research Topic.
